# The effect of orthodontic bracket pad shape on shear bond strength, an *in vitro* study on human enamel

**DOI:** 10.4317/jced.55121

**Published:** 2018-08-01

**Authors:** Nirav Patel, Prashanti Bollu, Kishore Chaudhry, Karthikeyan Subramani

**Affiliations:** 1 Roseman University of Health Sciences, College of Dental Medicine, Henderson, NV, USA

## Abstract

**Background:**

To evaluate the effect of bracket pad shape on shear bond strength (SBS) of orthodontic brackets bonded to human enamel.

**Material and Methods:**

One hundred and five extracted human maxillary permanent molars were divided into 7 groups of 15 specimens per group (n=15). Each group of teeth was bonded with 6 different shaped WildSmiles® brackets (Star, Heart, Soccer ball, Football, Flower, and Diamond) and GAC® rectangle shaped brackets. Shear debonding force was measured with an Instron universal testing machine using a knife-edged chisel 24 hours after initial bonding. Descriptive statistics (mean, standard deviation, and range) for each bracket pad shape was calculated. Analysis of variance (ANOVA) using SPSS software version 24.0 was performed with P-value set at 0.05. Post-Hoc Tukey analysis was used to analyze differences among groups. Differences in Adhesive Remnant Index (ARI) scores among groups were analyzed using Chi-square test.

**Results:**

Debonding force values (N ± SD) ranged from 205.51 ± 49.12 (Star) and 275.96 ± 69.05 (Soccer). SBS values (MPa ± SD) ranged from 13.34 ± 3.18 (Star) and 17.77 ± 6.94 (Rectangle). Even though intergroup comparison of SBS in Newtons revealed statistical significance (*p* = 0.014) between Star-Soccer and Star-Football group, it does not have any clinical significance since ranges of SBS of all groups are clinically acceptable. Analysis of ARI scores showed no significant differences in mode of bond failure among groups (*P* = 0.82).

**Conclusions:**

Orthodontic bracket pad shape has no effect on SBS and does not affect the mode of fracture pattern.

** Key words:**Shear bond strength, orthodontic bracket, bracket pad shape, orthodontic bracket base shape, adhesive remnant index.

## Introduction

Traditionally, the options for bracket style or appliance design were considerably limited for both the patient and the provider. Recently, the orthodontic market has experienced phenomenal growth in the development and production of orthodontic appliances that are designed to appeal to the patients. There are considerable differences in what patients indicate as the most attractive appliances, the one they would prefer to have (1). In 2009, Rosvall *et al.* (1) investigated to quantify laypersons’ assessments of attractiveness, acceptability, and value of orthodontic appliances. Orthodontic appliances were placed in a consenting adult, and digital images were captured, standardized, and incorporated into a computer-based survey. The survey displayed various images of orthodontic appliances for rating by a sample of adults (n=550). Subjects rated each image for: 1. attractiveness on a visual analog scale, 2. acceptability of placement of each appliance on themselves and their children, and 3. willingness to pay for each appliance for an adult or a child relative to a metal appliance standard. Rater reliability for the attractiveness, acceptability, and value ratings was assessed by rating 3 images twice. Attractiveness ratings were grouped in the following hierarchy of appliance types: alternative appliances such as clear trays and simulated lingual appliances > ceramic appliances > ceramic self-ligation appliances > all hybrid and stainless steel appliances. Acceptability ratings for all alternative and ceramic appliances were statistically equivalent, and statistically higher than those for other appliances.

Standard metal braces had the lowest acceptability rate of 55%. The willingness-to-pay value of appliances relative to a metal standard appliance ranged from $629 for lingual appliances to $167 for a hybrid self-ligation appliance. These findings show that a significant number of patients find commonly used appliances unattractive and unacceptable. Patients are willing to pay more money for appliances they deem more esthetic. In 2010, Walton *et al.* (2) investigated preferences and acceptability of orthodontic appliances in children and adolescents. Images of orthodontic appliances previously captured and standardized were selected and incorporated into a computer-based survey. Additional images of shaped brackets and colored elastomeric ties, as well as discolored clear elastomeric ties, were captured and incorporated onto existing survey images with Photoshop software. The survey displayed 12 orthodontic appliance variations to 139 children in 3 age groups: 9 to 11 years (n=45), 12 to 14 years (n=49), and 15 to 17 years (n=45). The subjects rated each image for attractiveness and acceptability. This study showed substantial differences in how children’s and adolescent’s preferences for orthodontic appliances differ from adults. Interestingly, in the 9-11 years and 12-14 years age groups, the appliance selected most frequently as the number 1 choice was some variation of WildSmiles® brackets at 44%. Since children and adolescents continue to make up the vast majority of orthodontic patients, understanding which appliances are acceptable to them will help practitioners meet their needs. One surprising finding of this study was the overall high rating of shaped brackets in all age groups. Acceptability for WildSmiles was highest in the youngest group at 70%, 25% higher than traditional ceramic brackets. Thus, it would appear that, if an orthodontic practice were to offer an alternative bracket to its standard appliance for children and adolescents, WildSmiles® brackets would most likely elicit more demand than a ceramic bracket.

WildSmiles® offer six shaped brackets: star, heart, soccer ball, flower, football, and diamond. They share many of the design similarities as the traditional metal braces other than bracket pad shape. Previous research studies determined that shear bond strength testing results can be influenced by a variety of factors, such as mesh wire gauge and mesh layer (3), bracket base surface area (4), bracket base design (5) and bracket pad shape (6). However, no research study has tested effect of bracket pad shape on shear bond strength on human enamel. Cucu *et al.* (4) investigated the *in vitro* shear bond strength of orthodontic brackets with 80- and 100-gauge mesh bases as well as mini and standard-size bases. They found no significant differences in the shear bond strength of any of the brackets compared. MacColl *et al.* (3) evaluated the effects of sandblasting bracket base mesh surfaces, reducing base surface area, and etching enamel with various acid types. They found that sandblasting and micro etching of foil-mesh bases increased the shear bond strength. In addition, they found no significant differences in the shear bond strength of bracket base surface areas between 6.8 mm2 and 12.4 mm2 but decreased when the surface area was at 2.4 mm2.

Knox *et al.* (5) evaluated different bracket base designs including 60-, 80-, and 100- gauge single mesh bases, a double mesh base, and integrated metal base. They concluded that the bonding agent significantly affects the shear bond strength and that particular base designs may allow improved adhesive penetration or improved penetration of the curing light. Pham *et al.* (6) in 2016 investigated the effect of bracket pad shape on shear bond strength on bovine enamel. The authors concluded that bracket base shape has an effect on shear bond strength. They found out that base shape with a pronounced tip at incisal base extension such as diamond, heart, star and soccer exhibited lower bond strength. There are no published studies investigating the effect of orthodontic bracket pad shape on bond strength on human enamel. The purpose of this study was to evaluate the effect of bracket pad shape on shear bond strength on human enamel.

## Material and Methods

-Test Samples

One hundred and five extracted human permanent molars were obtained and stored in 1:100 sodium hypochlorite solution (Clorox, Oakland, CA) prior to experiment. One-inch diameter PVC pipe (Lasco, Brownsville, TN) was used to mount teeth. Mounting jig was used to achieve proper tooth orientation with buccal surface perpendicular to the horizontal plane.

-Selection Criteria

The criteria for tooth selection included intact labial enamel, no cracks, no gross damage (under 10x magnification) and no caries.

Brackets and Bonding Materials

The total sample of 105 extracted permanent maxillary human molars were divided into 7 groups of 15 specimens (n=15). GAC® (Dentsply Sirona, York, PA) 0.022” slot rectangular base brackets were used for the control group, whereas remaining 6 groups consisted of WildSmiles® (WildSmiles, Omaha, NE) 0.022” slot brackets with six different bracket base shapes: Star, Heart, Soccer, Flower, Football, and Diamond.

-Bonding Procedure

1. Enamel surface was etched with 35% phosphoric acid Opal Etch (Ultradent, South Jordan, UT) for 15 seconds. It was then rinsed thoroughly with water and air dried until chalky appearance was observed.

2. Thin layer of Assure® (Reliance Orthodontic Products, Itasca, IL) bonding resin was applied to the etched enamel. It was dried with two bursts of compressed air and then light cured.

3. Light bond® (Reliance Orthodontic Products, Itasca, IL) bracket adhesive was applied to the bracket base, which was then placed on the enamel surface and firmly pressed on to the surface. Dontrix gauge was used to apply identical pressure (300 g) (7). Any excess of the adhesive was removed and light cured (Curing light XL 3000, 3M Unitek, Monrovia, CA) for 60 seconds from standardized distance of 6 mm.

4. Samples were stored in distilled water at 37oC degrees for 24 hours.

-Testing Procedure

**Figure 1:**
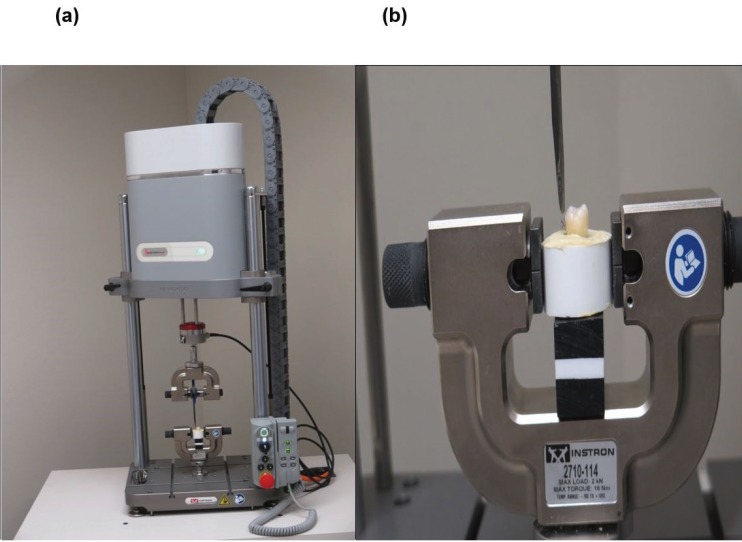
(a) Instron testing machine with sample held in position. (b) Instron attachment blade placed at the bracket ligature groove ready for testing at a crosshead speed of 1mm/min.

SBS testing was performed using an Instron universal testing machine (Fig. 1(a)) at a crosshead speed of 1 mm/min (8). Instron attachment blade was placed at the bracket base-tie wing interface (Fig. 1(b)) due to the differences in the geometrical shapes of bracket pads (9). Occluso-gingival force parallel with the bracket base was applied (10). The force in Newtons required to debond bracket was recorded. SBS in MPa was then calculated by dividing the debonding force (N) by its respective base surface area in square millimeters.

-Adhesive Remnant Index 

Artun and Bergland have used an adhesive remnant index (ARI) to evaluate the amount of adhesive left on the tooth after debonding (11). The debonded brackets were scored on a four-point scale by two independent examiners at two separate time points; using the following ARI index:

0: 100% of the adhesive remaining on the bracket

1: More than 50% of the adhesive remaining on the bracket

2: Less than 50% of the adhesive remaining on the bracket

3: No adhesive remaining on the bracket

Statistical Analysis

Descriptive statistics (mean, standard deviation, and range) for each bracket pad shape was calculated. Analysis of variance (ANOVA) using SPSS software version 24.0 (IBM, Chicago, IL) was performed with *P*-value set at 0.05. Post-Hoc Tukey analysis was used to investigate differences among different groups. Differences in ARI scores among different groups were analyzed using Chi-square test.

## Results

**Figure 2:**
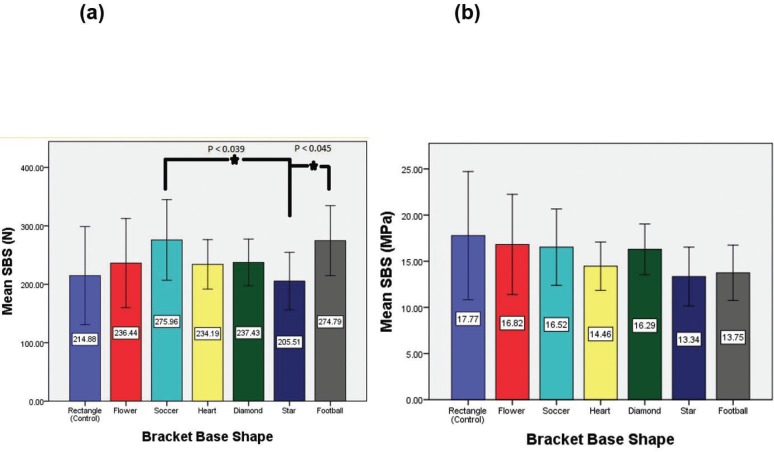
(a) Comparison of Mean SBS (N) with standard deviation of brackets with different base shapes. (b) Comparison of Mean SBS (MPa) with standard deviation of brackets with different base shapes.

Figure 2(a) represents mean Shear bond strength recorded in Newton. Debonding force values (N ± SD) ranged from 205.51± 49.12 (Range 113.20-280.20) (Star group), 214.88 ± 83.93 (Range 128.40-454.20) (Rectangle group) and 274.78 ± 59.93 (Range 173.60-403) (Football group), 275.96 ± 69.05 (Range 158.30- 378.50) (Soccer group). Heart (234.19 ± 42.38) (Range 149.80-295.30) and Diamond (237.43 ± 40.04) (Range 159.90-284.60) groups showed almost similar debonding force values. One way ANOVA showed significant differences in bond strength measurements between experimental groups (*P*= 0.014). Post-hoc inter-group comparison revealed significant differences between Star-Soccer and Star-Football groups.

Figure 2(b) represents mean SBS with SD in MPa± SD. Characteristic MPa values ranged from 13.34 ± 3.18 (Range 8.68-20.16) (Star group), 13.74 ± 2.99 (Range 7.37-37.5) (Football group) and 17.77± 6.94 (Range 10.62-37.57) (Rectangle group). Heart (14.46 ± 2.61) (Range 9.25-18.24), Diamond (16.28 ± 2.74) (Range 10.97-19.52), Soccer (16.52 ± 4.13) (Range 9.48-22.66), and Flower (16.81 ± 5.42) (Range 9.89-31.17) groups showed almost similar MPa values. One way ANOVA showed no significant differences in bond strength measurements between experimental groups (*P*= 0.078).

Frequency distribution with mean and standard deviation of the ARI scores are given in  Table 1. Analysis of ARI scores showed no significant differences in the mode of bond failure among groups (*P* = 0.82).

**Table 1:**
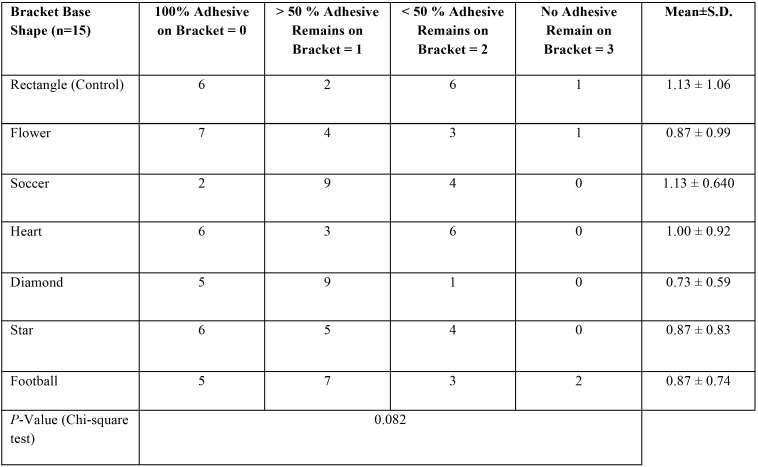
Adhesive Remnant Index Frequency Distribution Table with Mean Score ±SD.

## Discussion

The minimum bond strength required for clinical success is related to the forces of occlusion and not to the forces generated by an orthodontic arch wire (12). The use of a thin transducer to measure the maximum biting force during chewing by a patient on command has been reported that, in children with normal lower face heights between the ages of 6 and 11 years, this force is 49N and in adults 149 N (12). These results are similar to the values reported by another study where thick strain gauges were used (3). It would thus be reasonable to infer from these studies that bracket displacement forces may range from 49 to 149 N. Bond strengths have been measured by multiple testing types; most commonly shear, peel, tension and torsion. Tension and shearing tests are the most common methods of testing bracket bond strengths. Both are considered to provide similar and clinically comparable values. The shearing force created by mastication and occlusal forces, if greater than bond strengths, will result in bracket failure. It has been determined that clinically acceptable SBS ranges from 5.9-7.8 MPa (12). In the current study, all groups produced SBS values that clearly exceeded the clinically acceptable range. Mean SBS of control group in Newton was 214.88±83.93, whereas it ranges from 205-275 N for experimental groups. Even though intergroup comparison of shear bond strength in Newtons revealed statistical significance between Star-Soccer and Star-Football group, it does not have any clinical significance since ranges of SBS of all groups are clinically acceptable. However, higher shear bond strengths may be associated possible damage to the enamel surfaces. Therefore, it would be prudent to be more careful when debonding these brackets.

No significant differences in ARI scores were found between the experimental groups in our study. Examination of the tooth surface and bracket base after debonding indicates that resin may adhere either to the bracket base or to the tooth surface. Adherence to the bracket base is indicative of surface enamel removal during the debonding process, whereas adherence to the tooth suggests that enamel surface remained intact. Analysis of ARI scores indicates that most bond failures occurred at enamel-adhesive interface. Although final polishing of the teeth after debonding would appear to be the same after both types of resin fracture, these finding suggest that, if the majority of debonds occur at the enamel resin interface, the fluoride rich surface enamel in children from fluoridated areas has been compromised. As a result, clinicians would be well advised to consider topical fluoride regimens to restore this fluoride balance (3).

Pham *et al.* (6) investigated the effect of bracket pad shape on shear bond strength on bovine enamel. They concluded that bracket base shape has an effect on shear bond strength. They found out that base shape with a pronounced tip at incisal base extension such as diamond, heart, star and soccer exhibited lower bond strength. Difference in results between this study and present study may be attributed due to differences in bovine and human enamel and/or use of upper permanent human molars instead of incisors. The present study confirms the previous finding by Oesterle *et al.* (13) that the enamel bond to bovine teeth is 21% to 44% weaker than human enamel. Upper Molars were used in this research study due to potential difficulties in finding Maxillary incisors. Since surface area was accounted for in calculating MPa values, results may not be different for anterior teeth.

## Conclusions

• Orthodontic bracket base shape has no effect on shear bond strength

• Bracket base shape does not affect the mode of fracture pattern.

## References

[1] Rosvall MD, Fields HW, Ziuchkovski J, Rosenstiel SF, Johnston WM (2009). Attractiveness, acceptability, and value of orthodontic appliances. Am J Orthod Dentofacial Orthop.

[2] Walton DK, Fields HW, Johnston WM, Rosenstiel SF, Firestone AR, Christensen JC (2010). Orthodontic appliance preferences of children and adolescents. Am J Orthod Dentofacial Orthop.

[3] MacColl GA, Rossouw PE, Titley KC, Yamin C (1998). The relationship between bond strength and orthodontic bracket base surface area with conventional and microetched foil-mesh bases. Am J Orthod Dentofacial Orthop.

[4] Cucu M, Driessen CH, Ferreira PD (2002). The influence of orthodontic bracket base diameter and mesh size on bond strength. SADJ.

[5] Knox J, Hubsch P, Jones ML, Middleton J (2000). The influence of bracket base design on the strength of the bracket-cement interface. J Orthod.

[6] Pham D, Bollu P, Chaudhry K, Subramani K (2017). Comparative evaluation of orthodontic bracket base shapes on shear bond strength and adhesive remnant index: An in vitro study. J Clin Exp Dent.

[7] Stanford SK, Wozniak WT, Fan PL (1997). The need for standardization of test protocols. Semin Orthod.

[8] Klocke A, Kahl-Nieke B (2005). Influence of cross-head speed in orthodontic bond strength testing. Dental Materials.

[9] Klocke A, Kahl-Nieke B (2005). Influence of force location in orthodontic shear bond strength testing. Dent Mater.

[10] Klocke A, Kahl-Nieke B (2006). Effect of debonding force direction on orthodontic shear bond strength. Am J Orthod Dentofacial Orthop.

[11] Årtun J, Bergland S (1984). Clinical trials with crystal growth conditioning as an alternative to acid-etch enamel pretreatment. American Journal of Orthodontics.

[12] Reynolds IR (1975). A Review of Direct Orthodontic Bonding. British Journal of Orthodontics.

[13] Oesterle LJ, Shellhart WC, Belanger GK (1998). The use of bovine enamel in bonding studies. American Journal of Orthodontics and Dentofacial Orthopedics.

